# A case of stage IV gastric cancer with para-aortic lymph node metastasis showing pathological complete response after neoadjuvant chemotherapy

**DOI:** 10.1186/s40792-020-0788-1

**Published:** 2020-01-10

**Authors:** Yuki Katsura, Takehiro Okabayashi, Manabu Matsumoto, Kazuhide Ozaki, Yuichi Shibuya

**Affiliations:** 1 Department of Gastroenterological Surgery, Kochi Health Sciences Center, 2125-1 Ike, Kochi City, Kochi 781-8555 Japan; 2 Department of Diagnostic Pathology, Kochi Health Sciences Center, 2125-1 Ike, Kochi City, Kochi 781-8555 Japan

**Keywords:** Gastric cancer, Para-aortic lymph node metastasis, Conversion surgery, Pathological complete response

## Abstract

**Background:**

Stage IV advanced gastric cancer with para-aortic lymph node metastasis (PALM) is considered unresectable. Systemic chemotherapy is the treatment of choice for such tumors, while conversion surgery may be a treatment option in the case chemotherapy is effective but R0 resection is possible. We report a case of stage IV gastric cancer with PALM that showed pathological complete response (pCR) after neoadjuvant chemotherapy (NAC) using S-1, oxaliplatin, and trastuzumab (SOX+HER).

**Case presentation:**

A 69-year-old woman who was diagnosed with type 4 stage IV gastric cancer with PALM underwent five courses of NAC with the SOX+HER regimen. The primary tumor and the PALM shrank after treatment, suggesting that the NAC induced a partial response. We performed a total gastrectomy plus distal pancreaticosplenectomy with para-aortic lymph node dissection. Histological analysis revealed no remnant cancer cells in the primary tumor or the lymph nodes, confirming a pCR. The postoperative course was uneventful, and the patient was discharged on day 14 after the operation. S-1 was started as adjuvant chemotherapy, and the patient remains alive without recurrence 2 months after surgery.

**Conclusion:**

This case shows the possibility of conversion surgery after SOX+HER therapy for stage IV advanced gastric cancer with PALM.

## Background

Gastric cancer is the fifth most common cancer and the third leading cause of mortality among all cancers worldwide [1]. Stage IV gastric cancer with para-aortic lymph node metastasis (PALM) is considered an unresectable metastatic disease, and its prognosis remains poor after isolated surgical treatment [2]. The best clinical practice for patients with clinical PALM has remained controversial for over 10 years [3]. Preoperative chemotherapy was recently adopted by studies, and Japanese oncologists have reported an encouraging 5-year survival rate of 53% for gastric cancer with PALM treated by D2 gastrectomy with para-aortic lymph node dissection (PAND) after neoadjuvant chemotherapy (NAC) [4]. Therefore, according to fifth edition of the Japanese gastric cancer treatment guidelines, in cases of stage IV gastric cancer with limited numbers of PALM and Bulky N without other non-curative factors, surgical resection after NAC is suggested. However, developing a safe and standard D2 plus PAND protocol after chemotherapy remains challenging, and to date, only a few surgeons worldwide have performed it expertly. In the present case, we successfully performed radical surgery after the NAC, and the postoperative histological analysis showed a pathological complete response.

## Case presentation

**Fig. 1:**
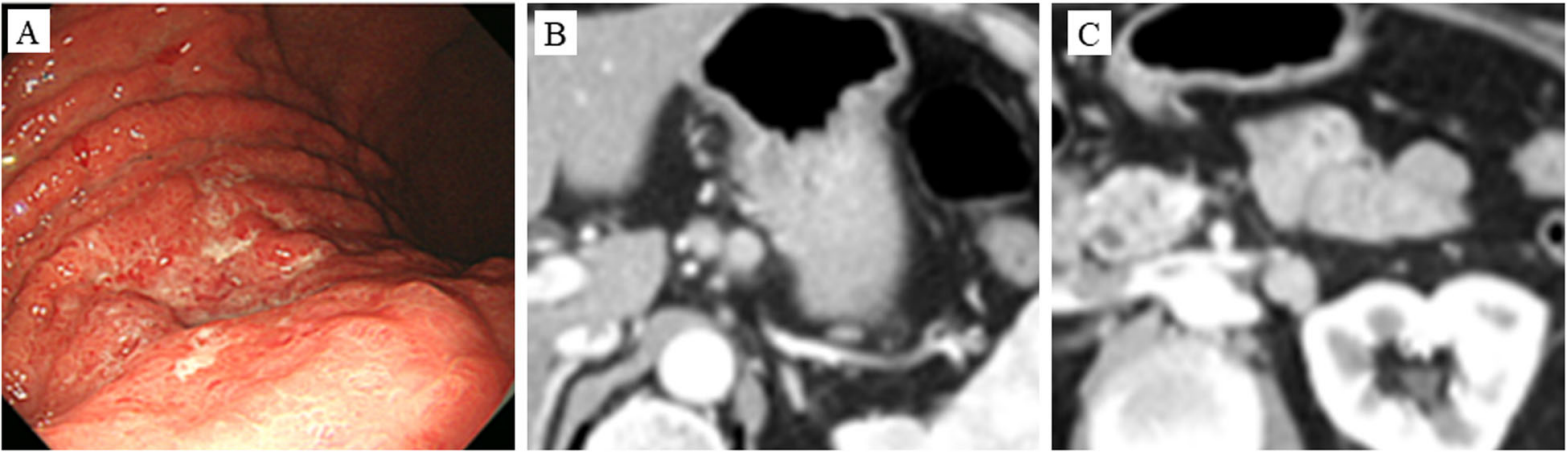
Esophagogastroduodenoscopy (EGD) findings and computed tomography (CT) findings before chemotherapy. **a** EGD before chemotherapy revealed Borrmann type 4 cancer at the greater curvature from the upper to middle part of the gastric corpus. Abdominal CT before chemotherapy showed swollen regional lymph nodes (**b**) and para-aortic lymph nodes (**c**)

**Fig. 2:**
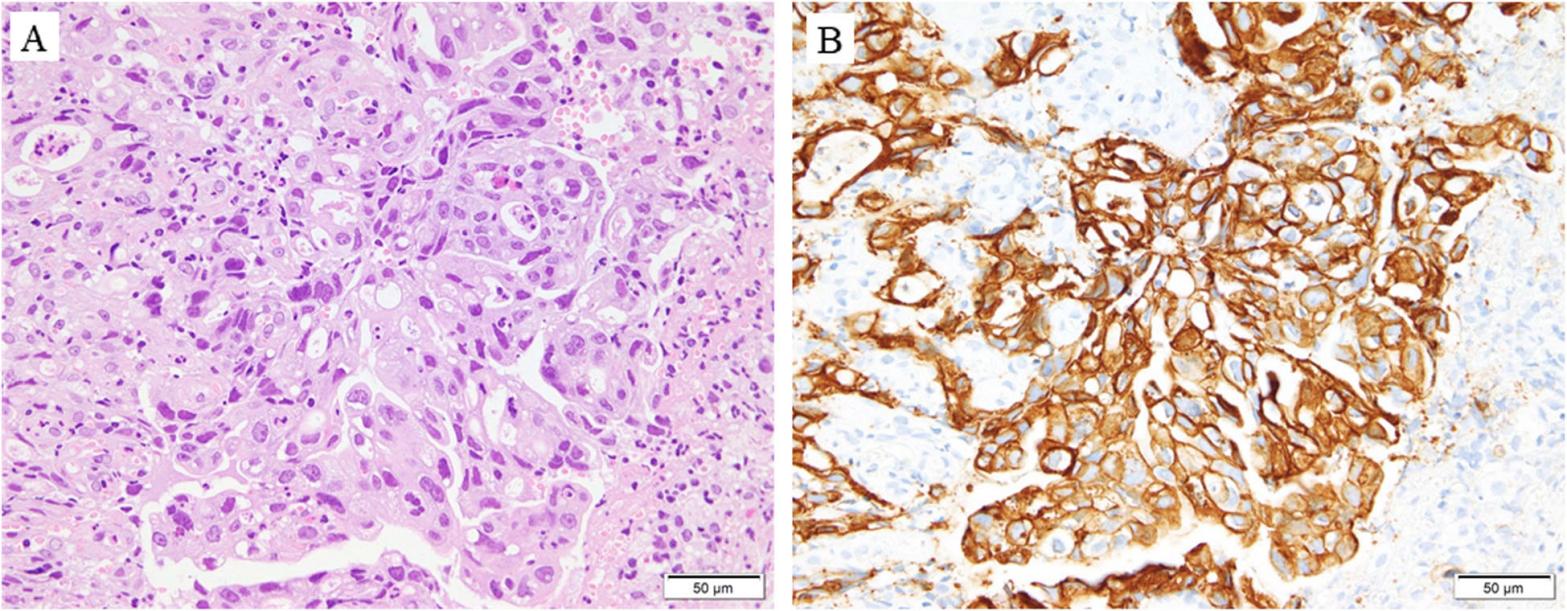
**a** Hematoxylin and eosin staining in the biopsy specimen confirming poorly differentiated adenocarcinoma. **b** HER2 immunohistochemical staining in the biopsy specimen confirming strong expression of HER2 in the tumor cell

A 69-year-woman with a 1-month history of epigastric pain was referred to Kochi Health Sciences Center. A physical examination revealed no palpable masses in the abdomen or superficial lymphadenopathy. Blood tests showed normal hepatobiliary and pancreatic enzyme levels, renal function, and carcinoembryonic antigen levels. However, the patient’s serum carbohydrate 19-9 levels were increased (193 U/mL, normal range, < 37 U/mL). EGD revealed diffuse gastric wall thickening, hypertrophy of the mucosal folds, and irregular deep ulceration at the greater curvature from the upper to middle part of the gastric corpus (Fig. 1a). Abdominal computed tomography (CT) revealed a thickened gastric wall and swollen lymph nodes along with the lesser curvature (Fig. 1b), celiac artery, splenic artery, and PAN (nos. 16a2, 16b1; Fig. 1c). No distant metastasis or ascites were identified. Biopsy confirmed a poorly differentiated adenocarcinoma (Fig. 2a) and a tumor cell cluster with strong basolateral membranous reactivity to HER2 immunohistochemical staining (Fig. 2b). The clinical diagnosis was cT4aN2M1 (LYM) stage IV according to the 15th edition of the Japanese Classification of Gastric Carcinoma, and the S-1, oxaliplatin, and trastuzumab (SOX+HER) chemotherapy was initiated for the treatment of unresectable advanced gastric cancer (S-1, 100 mg/body/day days 1–14; oxaliplatin, 130 mg/m^2^ day 1; trastuzumab, 6 mg/kg day 1).

**Fig. 3:**
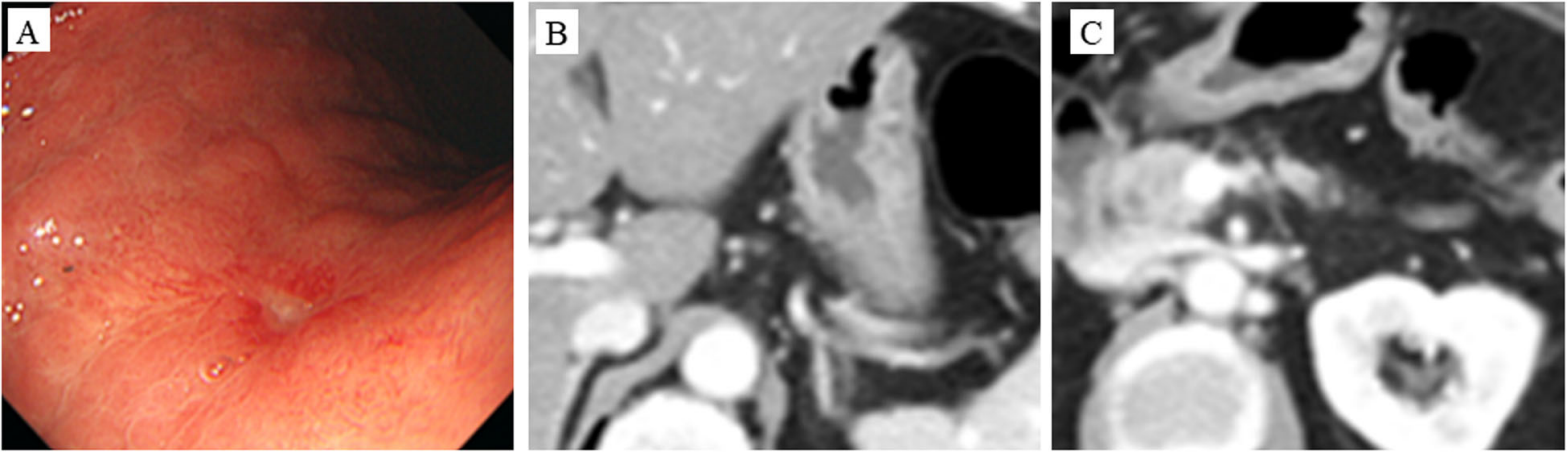
Esophagogastroduodenoscopy (EGD) and computed tomography (CT) findings after chemotherapy. **a** EGD after chemotherapy detected a scar lesion instead of a tumor. Abdominal CT after the five courses of S-1, oxaliplatin, and trastuzumab revealed regression of the regional lymph nodes (**b**) and para-aortic lymph nodes (**c**)

**Fig. 4:**
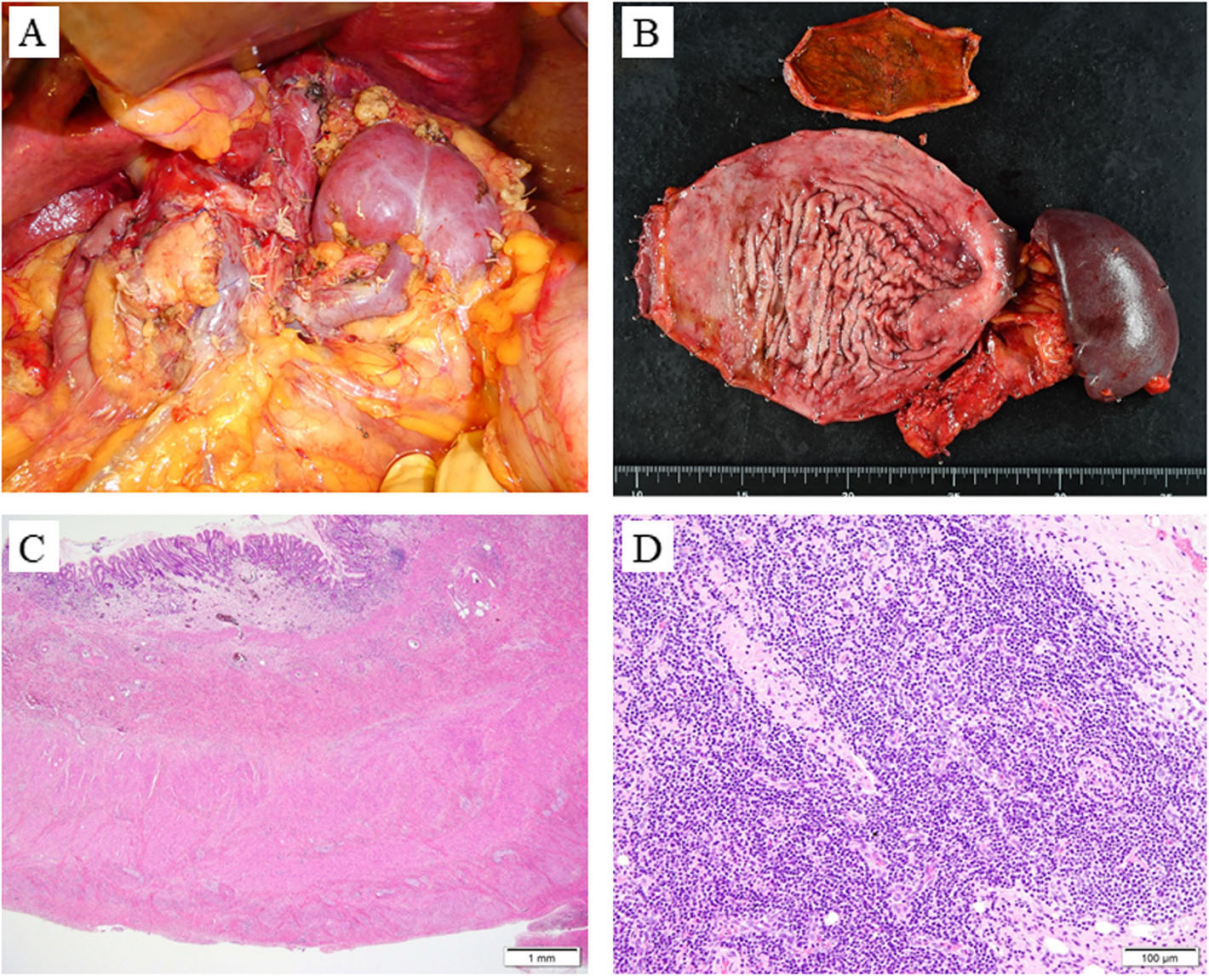
**a** Intraoperative image taken after total gastrectomy plus distal pancreaticosplenectomy with para-aortic lymph node (PAN) dissection. **b** Surgically resected specimen. Macroscopic observation indicated complete regression of the primary lesion. **c** Histopathological findings of the resected specimen. Atrophy and fibrosis of the gastric mucosa in the lesion area were demonstrated with no infiltration of cancer cells at any site. **d** There were no signs of residual cancer cells in the resected PAN

Follow-up endoscopy after five courses of SOX+HER therapy detected a scar lesion instead of a tumor (Fig. 3a). Furthermore, an abdominal CT scan revealed a smaller primary lesion, regional lymph node (Fig. 3b), and PAN (Fig. 3c). We believed that R0 resection with intent to cure was possible and planed the conversion surgery. Laparotomy examinations showed no peritoneal dissemination, and peritoneal lavage cytology revealed no cancer cells in the abdominal cavity. We performed a total gastrectomy plus distal pancreaticosplenectomy with para-aortic lymph node dissection and Roux-en-Y reconstruction (Fig. 4a). The postoperative course was uneventful, and the patient was discharged 14 days after surgery. Macroscopically, the tumor was not palpable as an elastic hard mass (Fig. 4b). The histological findings of the surgically resected specimen revealed atrophy and fibrosis of the gastric mucosa in the lesion area accompanied by inflammatory cell infiltration, and there was no clear infiltration of cancer cells at any site (Fig. 4c). Also, no remnant cancer cells in the lymph nodes, including PAN, were detected (Fig. 4d). Pathological grading indicated that the resected lesion was grade 3, confirming a pathological CR (ypT0N0M0). Adjuvant chemotherapy with S-1 was initiated after surgery. At 2 months after surgery, she remains alive without recurrence.

## Discussion

Chemotherapy is considered the primary treatment choice for stage IV gastric cancer, but its prognosis remains poor [2, 5]. Surgery is not routinely recommended except for palliative reasons. Under some conditions, the treatment of clinical stage IV gastric cancer with a single incurable factor, such as PALM, positive lavage cytology, and sole liver metastasis, may be controversial [6]. Lymph node metastases and positive cytology on peritoneal washing as unresectable factors are related to better prognoses after conversion surgery when a partial or complete response to chemotherapy was observed [6]. However, there are no evidence-based NAC regimens for advanced gastric cancer, and clinical trials are now ongoing (JCOG1509).

In the present case, we selected SOX plus trastuzumab therapy as the primary treatment because trastuzumab with cisplatin plus capecitabine or S-1 is the recommended regimen according to several phase II studies in HER-2-positive advanced gastric cancer [7–9]. Takahari et al. reported the efficacy and safety of combination therapy consisting of trastuzumab plus SOX for patients with HER-2-positive advanced gastric cancer [10]. Indeed, another determining factor is the detection of the best timing to operate (or not operate). Surgery generally occurs when the tumor decreases in size and before it develops drug resistance. For this determinant decision-making step, cooperation between oncologists and surgeons is mandatory for the general management of patients (and not the tumor alone). In the current series, a CT scan showed a partial response against the primary tumor and lymph node metastasis, which made us choose conversion surgery, whereas the efficacy of conversion surgery remains unclear [6].

Due to the fact that suspicious lymph node enlargement can result from inflammatory lymphadenopathy or malignancy, patients with radiologically overt PALM may have entirely different pathological stages (stage IV or not) that will require completely different treatment strategies. However, the pathological diagnosis of enlarged PAN is difficult. PAN biopsy is an invasive and technically difficult manipulation; thus, it is not typically used for clinical diagnosis of PALM in most institutes. In addition, positive lymph nodes will disappear or shrink after preoperative treatment, which makes it difficult to re-biopsy the original nodes during follow-up. In our series, we found that PALM disappeared with isolated PAN as CR pathologically. Whether surgical resection is needed for stage IV gastric cancer remains controversial [11, 12]. PALM is classified as a relatively early type in stage IV gastric cancer, is associated with a lower tumor burden than other organ and peritoneal metastases, and could be the most suitable surgery type among all types of stage IV gastric cancer [6]. We performed total gastrectomy plus distal pancreaticosplenectomy with para-aortic lymph node dissection in terms of the lymphatic metastasis system of gastric cancer. In the present case, because swollen lymph nodes along with the lesser curvature, celiac artery, splenic artery, and PAN were detected, the en bloc resection of primary lesion and metastatic lymph node tissue by total gastrectomy plus distal pancreaticosplenectomy with para-aortic lymph node dissection was necessary to remove the cancer cells without exposure.

## Conclusion

In conclusion, here, we described a case of successful conversion surgery with SOX+HER therapy for stage IV gastric cancer with PAN metastasis. Neoadjuvant therapy with SOX+HER and conversion surgery might be an effective treatment for stage IV gastric cancer with PAN metastasis. Further evidence and prospective clinical trials are required to establish the optimal strategy for stage IV gastric cancer with PAN metastasis.

## Data Availability

All data generated or analyzed during this study are included in the published article.

## Abbreviations

EGD: Esophagogastroduodenoscopy
NAC: Neoadjuvant chemotherapy
PALM: Para-aortic lymph node metastasis
PAND: Para-aortic lymph node dissection
pCR: Pathological complete response
SOX+HER: S-1, oxaliplatin, and trastuzumab
